# Effects of transitional health management on adherence and prognosis in elderly patients with acute myocardial infarction in percutaneous coronary intervention: A cluster randomized controlled trial

**DOI:** 10.1371/journal.pone.0217535

**Published:** 2019-05-31

**Authors:** Qing Wu, Dandan Zhang, Qi Zhao, Lin Liu, Zhisong He, Yan Chen, Hui Huang, Yunyin Hou, Xiaofang Yang, Jie Gu

**Affiliations:** 1 Nursing Department, The First Affiliated Hospital of Soochow University, Suzhou, China; 2 Department of Radiotherapy, The First Affiliated Hospital of Soochow University, Suzhou, China; 3 Department of Cardiology, The First Affiliated Hospital of Soochow University, Suzhou, China; 4 Nursing Department, the 2^nd^ Affiliated Hospital of Soochow University, Suzhou, China; Universidad Miguel Hernandez de Elche, SPAIN

## Abstract

**Purpose:**

This study aimed to assess the effects of transitional health management on adherence and prognosis in elderly patients with acute myocardial infarction undergoing percutaneous coronary intervention.

**Methods:**

We conducted the trial from June 2016 to December 2016. A total of one hundred and fifty patients with acute myocardial infarction after PCI who met the inclusion criteria were randomly divided into an experimental (n = 75) group and a control (n = 75) group. The participants in the experimental group received transitional health management for three months. The two groups of patients were evaluated for treatment adherence, quality of life, clinical indicators, adverse cardiovascular events and statistics regarding readmission rates at baseline and 6 months after discharge.

**Results:**

Compared with the controls, patients in the intervention group demonstrated better medication adherence, reexamination adherence, healthy lifestyle and clinical indicators (all P<0.05) and lower rates of adverse cardiovascular events and readmission (all P<0.05).

**Conclusion:**

Transitional health management effectively improved adherence in elderly patients with acute myocardial infarction after PCI, ameliorated clinical indicators, and effectively reduced the incidence of adverse cardiovascular events and readmission rates. Transitional health management was an effective intervention for PCI patients after discharge.

## Introduction

Cardiovascular diseases are the leading cause of death globally[1]. Approximately 17 million people die of cardiovascular disease each year, and 80% of deaths occur in less developed countries[2]. It is expected that by 2020, the danger of cardiovascular disease will rise to first place and will become one of the most important public health problems in China[3]. Coronary heart disease is the most common type of cardiovascular disease, accounting for 67.1% of the death rate[4]. The average cost of hospitalization also tops all medical diseases. At present, there are approximately 290 million patients with cardiovascular diseases in China, including 2.5 million patients with myocardial infarction which known as a heart attack and occurring when blood flow decreases or stops to a part of the heart, causing damage to the heart muscle [5]. In recent years, our country’s acute myocardial infarction (AMI) death rates have steadily increased. From 2002 to 2015, the mortality rate from coronary heart disease in China increased by nearly three-fold[5].

In recent years, along with social and economic developments, the aging of society has accelerated, and the incidence of AMI in the elderly has increased year by year[6]. Percutaneous coronary intervention (PCI) can maximize the restoration of coronary blood flow and promote the recovery of cardiac function[7]. Furthermore, an increasing number of elderly patients with coronary heart disease are selecting interventional therapy. Many studies have shown that PCI has the advantages of minimal trauma, fast recovery and fewer complications[8,9]. Every year, the rate of the number of patients undergoing PCI increases by 30%-40% in China[10]. PCI has been widely considered as a treatment measure that is safe and effective[11].

However, in China, most patients with AMI are discharged in 1 to 2 weeks when they are very stable[12]. Elderly patients have the unique characteristics of old age, physiological deterioration, and many complications; thus, if health management is not strengthened after PCI, there is still the possibility of coronary restenosis. It is common for people to stop taking their medication completely or to take it less frequently than prescribed[13]. Furthermore, studies have shown that nearly an estimated one-third of patients do not adhere to the blood pressure or lipid-lowering treatments that are prescribed following a myocardial infarction (secondary prevention)[14].

In addition, studies[15] have shown that 81.7% of patients believe that after discharge, the medical staff will continue to provide relevant services. This indicates that most patients hope to receive care and follow-up from the hospital after they leave the hospital[16].

If we can strengthen health management during the transition period, it will have important clinical significance and will help ensure that patients experience the maximum therapeutic effect of their treatment and that treatment efficacy and patient prognosis after discharge will be enhanced.

An intervention that used an adolescent peer support group was found to be an effective way to improve adherence to ART and improve patients’ self-image, with previously fearful adolescents perceived as becoming more confident and outgoing[17].

Health management is the process of using information and medical technology so that individual patients can engage in personalized health management[18], based on the health care and the medical treatment received. Health management enables healthy and less healthy people to develop a good, orderly and healthy lifestyle by promoting health, motivating individual enthusiasm, reducing risk factors, and thus decreasing the incidence of disease. Studies have shown[8] that the period from 2–6 months after discharge is critical for patients to transition from the hospital to a community clinic or to care with family members for continuous treatment or health recovery. If we can strengthen health management during the transition period, it will have important clinical significance for the efficacy of treatment and the prognosis of the discharged patients.

Based on the above, this study used a single-blind (patient), randomized, controlled method to conduct a 3-month transitional health management program for patients undergoing successful coronary stent implantation. Therefore, the main goal of our study was to determine whether the health management intervention improved compliance with the treatment, ameliorated clinical indicators and reduced cardiovascular events.

## Materials and methods

### Study design

A randomized controlled trial was conducted over 12 weeks to assess the effects of a 3-month transitional period health management intervention on treatment adherence, quality of life and prognosis in elderly patients with acute myocardial infarction undergoing PCI. This study was registered at http://www.chictr.org.cn/edit.aspx?pid=28366&htm=4 (ChiCTR1800016678, supplemental clinical registration). The authors confirm that all ongoing and related trials for this drug/intervention are registered.

### Ethics

This study was approved by the ethical committee of the First Affiliated Hospital of Soochow University(2016060,) and was accorded to the requirements of the Declaration of Helsinki[19]. All study participants signed informed consents.

### Participants

The sample size estimation was carried out using G*Power 3.1.9.2 software of two population means formulae[20]. According to other similar studies, the effect rates of the control group and the intervention group were calculated with a 0.447 effect size at a 5% significance level and a power of 0.8. This means that 128 participants were needed in each group for this study. However, anticipating a 20% potential attrition rate, additional participants were considered. Therefore, the sample size was expanded to include 70 participants in each group.

Thus, a total of 140 eligible participants were recruited and screened from the two cardiovascular inpatient departments of the Teaching Hospital in Suzhou from June 2016 to December 2016. In this trial, the inclusion criteria for participation were 1) having been diagnosed with myocardial infarction, having stable vital signs and being arranged for PCI for the first time; 2) being 60 years old or above; 3) having the ability to communicate sufficiently (in Mandarin) to understand the education program; and 4) having no other serious complications that would interfere with the program (e.g., tumor, uremia).

Participants who met the inclusion criteria and agreed to participate were invited to join the program. A screening process was strictly performed to determine the eligibility[21].

### Procedure

**Fig 1:**
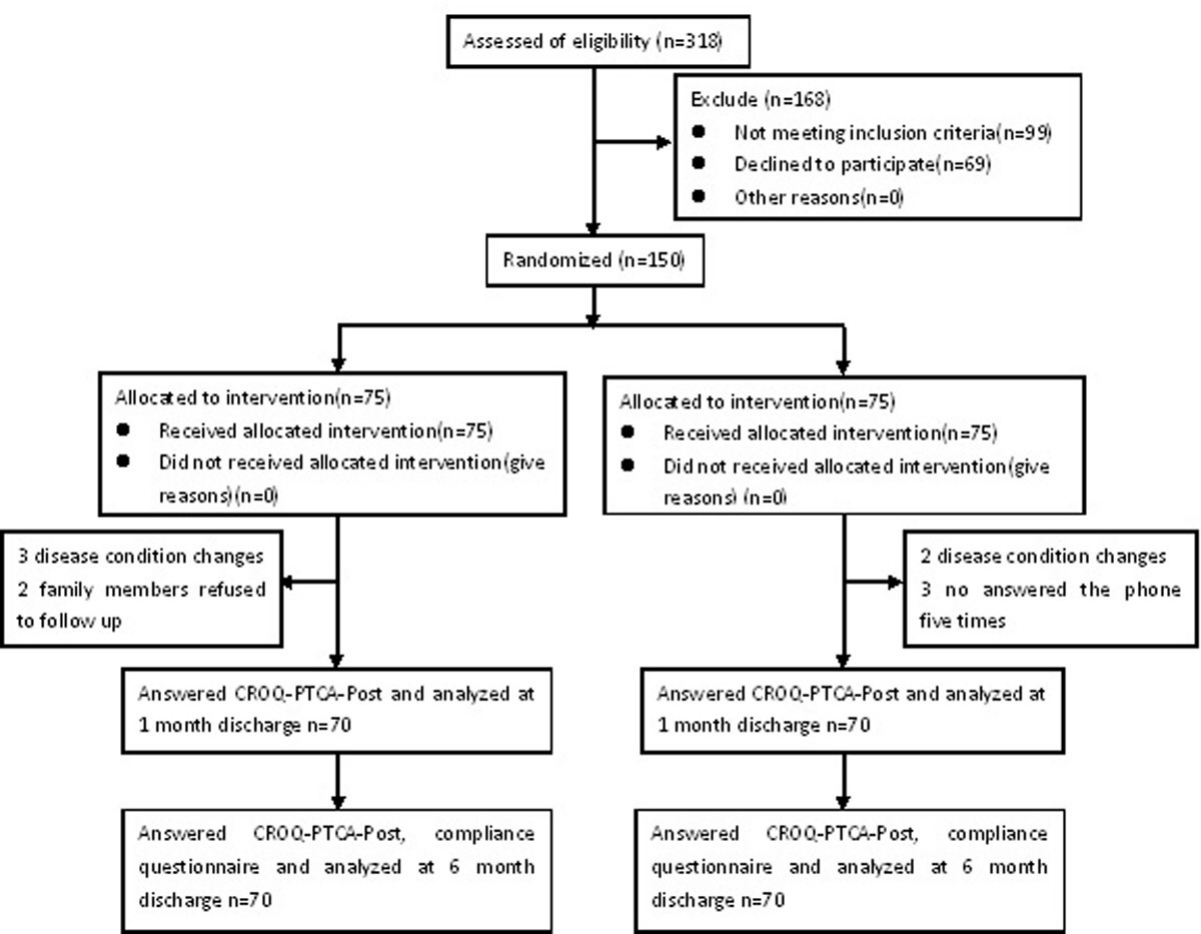
Consort diagram showing the flow of the entire study.

The study lasted for 6 months at all facilities, beginning on 1 June 2016 and ending on 31 December 2016. A total of 318 elderly patients with acute myocardial infarction undergoing PCI were identified from two hospitals in Suzhou. Of these potential participants, 99 were not interested in participating. Therefore, 219 people were screened for eligibility. Among them, 69 elderly patients with acute myocardial infarction were excluded, leaving only 150 (47.2%) participants who were eligible and agreed to participate in this study. Fig 1 shows the flow of participants during the entire implementation of the health management intervention (S1 and S2 Files).

The participants were numbered based on the order of their visit and were divided into the intervention group and the control group by the SPSS program. To avoid sample contamination (two groups may communicate with each other in the same ward), one of the subjects was adjusted into the intervention group when the intervention group and the control group were in the same ward. Next, one of the subjects was adjusted into the control group[22]. All participants in the intervention group received the health management intervention for 3 months. All participants in the control group received standard nursing care, including smoking cessation, sports, and diet. The patients in the control group were given telephone follow-up each month after discharge to obtain information[23].

#### The health management intervention program

On the basis of relevant literature at home and abroad, the research team completed the first draft of the “Handbook of Transitional Health Management after PCI” (S3 File). Then, the two deputy chief physicians of cardiovascular medicine and two experienced health management professionals reviewed and revised the draft after 3 rounds, which was the second draft. Finally, five patients without dyslexia who met the criteria included in the study were invited to read and suggest corrections to complete the final draft. The handbook was distributed to patients and their families in the intervention group on the first postoperative day after PCI and explained one by one, repeatedly emphasizing the precautions during the transition period. After discharge, two follow-up methods were adopted: telephone and home visit. Each patient was assigned a health management file and had a personalized intervention outline and follow-up timetable developed. We implemented the program strictly. The follow-up content included assessing the patient's learning needs; emphasizing the importance of maintaining a good lifestyle; and helping patients to strengthen their self-management skills, such as quitting smoking and drinking, engaging in regular work and rest, consuming less salt and oil and eating a lighter diet, and participating in aerobic exercises such as walking and swimming. In addition, drug guidance was provided, and patients were encouraged to review the instructions regularly. Follow-up time was 3 months for a total of 12 weeks. According to the Ebbinghaus Forgetting Curve (Fast, Slow, Slow) feature[24], the telephone was used to follow up once a day after discharge. If no new problems occurred for 3 days, phone calls were changed to 2 times a week for 1 month. Then, follow-up was carried out once a month up to 12 weeks. If the patients developed new problems during follow-up, the above intervention would be repeated for up to 12 weeks. Follow-up visits were performed in the second week after discharge and did not exceed 30 minutes. Telephone follow-ups were restricted to 10 minutes. During the follow-up, we addressed the patient's doubts and evaluated the effectiveness of the intervention. For different patients, the rehabilitation process required for their disease was not the same and was constantly changing. Therefore, the plan was adjusted accordingly to reflect the actual situation and to ensure that the measures were put in place. The protocol is presented in files S4 and S5 Files.

#### Outcome measures

Demographics, questionnaire results and clinical data were collected by researcher-designed questionnaires during the intervention program. The demographic data (age, sex, marital status, education level, income) and clinical characteristics (number of diseased vessels, complications, smoking status, blood pressure, blood lipids, body mass index) were collected as baseline data. The primary outcomes of our study were treatment adherence, quality of life and adverse cardiovascular events. The secondary outcomes were clinical indicators and readmission rate. Participants in both the intervention and control groups responded to the CROQ-PTCA-Post three times and the treatment compliance questionnaire two times, either electronically or in print (>70% in Chinese), over the course of the study. Responses were scored according to established methods. All questionnaires were distributed by the same person, one by one. If there was doubt about the questionnaires, an unbiased interpretation would be given by the investigator.

Quality of Life was measured using the CROQ-PTCA-Post assessment. The CROQ-PTCA-Post was established by Sara Schroter in 2004[25]. The Chinese version of CROQ-PTCA-Post used in this study was translated by Songmei Cao[26]. It is composed of 47 self-reported items and 6 dimensions. The dimensions are symptoms, somatic function, psychosocial function, cognitive function, satisfaction with treatment, and adverse reactions. The scoring method of the scale is as follows: the original score is calculated according to the item value of each item in the questionnaire, in which items 7, 24 and 35 are not scored. The Chinese version has been validated, and the Cronbach's alpha coefficient of the dimensions was 0.80~0.94; the test-retest reliability was 0.83~0.94. The content validity was 0.8[26].

The treatment compliance questionnaire was provided by Liu Yan, Beijing Union Medical College Hospital, and it contains 14 items covering three dimensions: medication compliance, review compliance and lifestyle compliance[27]. Among them, the dimension of medication compliance is set to 4 questions, and each question is calculated using a 4-point system, including impossible, occasionally, basically and completely. If the subject cannot answer a question at all, he will get a score of 1. If the subject answers all of the questions, he will get a score of 4. A total score of 16 indicates good compliance, while a score below 16 indicates poor compliance. The Chinese version has been validated, the Cronbach's alpha coefficient of the dimensions was 0.85, and the test-retest reliability was 0.81[27].

#### Data analysis

Data were analyzed by another researcher who was blinded to the allocation using the PASW Statistic 17.0 Program (SPSS Inc.: Chicago, IL, USA). Measured data were expressed as the mean±standard deviation ($\bar{\text{x}}$±s). The t-test was used for the normally distributed data, and the Mann-Whitney U test was used for the skewed data between groups. For the quality of life analysis, multiple linear regression was used to adjust for other confounding factors. Comparisons between groups were performed using the chi-square test or Fisher's exact square test for categorical variables. Cumulative survival curves of cardiovascular events between groups were constructed using the Kaplan-Meier method. The log-rank test was used to compare cumulative event-free survival curves between groups. Univariate and multivariate Cox proportional hazard regression models were used to examine the effects of transitional health management intervention on cardiovascular events. The variables of age, sex, marital status, education level, income, endovascular stents, comorbidities, and smoking were the adjusted variables in the multivariate Cox model. For all analyses, missing data were transformed by mean imputation[28]. Throughout the analysis, a p-value of less than 0.05 was considered statistically significant.

## Results

### Participant characteristics

Table 1 outlines the characteristics of the 140 participating facilities. Baseline comparisons between the intervention and control groups showed that there was no significant difference at the pretest period (all P>0.05) (Table 1). At baseline, the mean age of the participants was 68.6 (SD 5.8) for the control group and 70.2 (SD 6.2) for the intervention group (P = 0.171); a majority of patients were male (70%). In addition, a majority of patients only had a junior high school education level or below (79%). In total, 75% of the patients had more than two endovascular stents, and 77% of the patients had complications.

**Table 1:** Baseline characteristics of participants according to demographic data and clinical characteristics by groups (n%/$\bar{\text{x}}$±s/Q_2_(Q_1_-Q_3_)).

Characteristic		Intervention group (n = 70)	Control group(n = 70)	Total(n = 140)	*P* Value
**Age**^**a**^		70.2±6.2	68.6±5.8	69.4±6.1	0.171
**Sex**^**b**^	**Male**	48 (68.6%)	50 (71.4%)	98(70%)	0.712
	**Female**	22 (31.4%)	20 (28.6%)	42 (30%)	
**Marital status**^**b**^	**Married**	47 (67.1%)	54 (77.1%)	101(72.1%)	0.187
	**Single**	23 (32.9%)	16 (22.9%)	39 (27.9%)	
**Education level**^**c**^	**Junior high school**	55 (78.5%)	56 (80.0%)	111 (79.3%)	0.782
	**Senior high school**	11 (15.7%)	9 (12.9%)	20 (14.3%)	
	**College**	4 (5.7%)	5 (7.1%)	9 (6.4%)	
**Income**^**c**^	**<1000**	5(7.1%)	8(11.4%)	13 (9.3%)	0.765
	**1000~2000**	41(58.6%)	39(55.7%)	80 (57.1%)	
	**>2000**	24(34.3%)	23(32.9%)	47 (33.6%)	
**Endovascular Stents**^**b**^	**1**	15 (21.4%)	20 (28.6%)	35 (25.0%)	0.499
	**2**	34 (48.6%)	34 (48.5%)	68 (48.6%)	
	**≥3**	21 (30.0%)	16 (22.9%)	37 (26.4%)	
**Comorbidity**^**b**^	**Yes**	56 (80.0%)	52 (74.3%)	108 (77.1%)	0.581
	**No**	14 (20.0%)	18 (25.7%)	32 (22.9%)	
**Smoking**^**b**^	**Never**	11 (15.7%)	8 (11.4%)	19 (13.6%)	0.472
	**Before**	29 (41.4%)	25 (35.7%)	54 (38.6%)	
	**Yes**	30 (42.9%)	37 (52.9%)	67 (47.8%)	
**Systolic blood pressure**^**d**^		127.5(110.0–138.0)	130(120.0–140.0)		0.095
**Diastolic blood pressure**^**d**^		75.0(70.0–84.0)	80(70.0–86.25)		0.058
**Total cholesterol**^**a**^		4.21±1.33	4.43±1.24	-1.402	0.163
**Triglyceride**^**a**^		1.82±1.61	1.85±1.32	-0.114	0.912
**High-density lipoprotein cholesterol**^**a**^		1.15±0.34	1.21±0.49	-1.119	0.263
**Low-density lipoprotein cholesterol**^**a**^		2.49±1.09	2.78±0.98	-1.803	0.060
**Body mass index**^**a**^		23.34±2.85	24.66±4.32	-0.864	0.412
**Left ventricular ejection fraction**^**a**^		42.56±8.70	42.61±10.42	42.58±9.57	0.968

^a^Independent t-test was used to compare the means of two groups for a normally distributed data

^b^Chi-square test was used to compare the proportion of two groups for categorical data

^c^Fisher's exact (2-sided) test was used to compare the proportion of two groups for categorical data (if the expected value of each cell was less than five)

^d^Mann-Whitney U test was used to compare the medians of two groups for a skewed data

### The effects of the transitional health management program on treatment compliance and quality of life

Before the experiment, there was no significant difference in treatment adherence and quality of life between the intervention group and the control group (P>0.05). At the posttest period, the chi-square test was used, and there was a statistically significant difference between the two groups in terms of treatment adherence (P<0.05), except for the sport compliance dimension (Table 2).

**Table 2:** Comparison of treatment adherence between two groups (n%).

Variable	Before the intervention				6 months after discharge			
	Intervention group (n = 70)	Control group (n = 70)	χ^2^	*P* Value	Intervention group (n = 70)	Control group (n = 70)	χ^2^	*P* Value
**Medication compliance**	18(25.7)	19(27.1)	0.037	0.848	64(91.4)	55(78.6)	4.538	0.033*
**Review compliance**	27(38.6)	32(45.7)	0.732	0.392	57(81.4)	39(55.7)	10.739	0.001**
**Control complications**	22(31.9)	21(30.0)	0.058	0.810	53(75.7)	39(55.7)	6.214	0.013*
**No smoking**	23(32.9)	21(30.0)	0.133	0.716	47(67.1)	28(40.0)	10.367	0.001**
**No drinking**	14(20.0)	22(31.4)	2.393	0.122	42(60.0)	29(41.4)	4.830	0.028*
**Low fat diet**	11(15.7)	8(11.4)	0.548	0.459	57(81.4)	30(42.9)	22.134	0.000**
**Low salt diet**	7(10.0)	10(14.3)	0.603	0.438	57(81.4)	44(62.9)	6.007	0.014*
**Sport compliance**	15(21.4)	18(25.7)	0.648	0.421	33(47.1)	22(31.4)	3.624	0.057

*P<0.05

**P<0.01

Mann-Whitney U tests suggested that the total scores for quality of life significantly changed across the three time points. (Table 3). At 1 month after discharge, the dimensions of physical function, psychosocial function, cognitive function, and treatment satisfaction in the intervention group were better than those in the control group (P<0.05, Table 3). At 6 months after discharge, the scores of all dimensions on quality of life were higher in the intervention group than in the control group (P<0.05, Table 3). The variables of age, sex, marital status, education level, income, endovascular stents, comorbidities, and smoking were the adjusted variables in the multiple linear regression (Table 4 and S6 File).

**Table 3:** Comparison of quality of life between two groups (Q_2_(Q_1_-Q_3_)).

Variable	Before the intervention			1 month after discharge			6 months after discharge		
	Intervention group (n = 70)	Control group (n = 70)	*P* Value	Intervention group (n = 70)	Control group (n = 70)	*P* Value	Intervention group (n = 70)	Control group (n = 70)	*P* Value
**Symptoms**	17.86(12.50–26.79)	15.46(10.41–20.52)	0.081	100.00(82.13–100.0)	83.01(73.21–100.0)	0.093	100.00(100.0–100.0)	100.00(81.97–100.0)	0.002**
**Physical function**	21.88(0.0–31.25)	15.63(4.69–25.00)	0.250	93.75(87.5–100.0)	93.75(87.50–93.75)	0.012*	100.00(93.75–100.0)	93.75(93.75–100.0)	0.004**
**Psychosocial function**	25.00(3.57–35.71)	17.86(8.93–44.64)	0.972	54.46(46.43–60.71)	37.50(32.14–46.42)	0.000**	75.00(62.50–85.71)	60.71(50.0–75.0)	0.007**
**Cognitive function**	60.00(40.0–73.33)	60.00(40.0–60.0)	0.078	73.33(60.0–86.67)	60.00(60.00–60.00)	0.000**	80.00(60.0–100.0)	80.00(60.0–80.0)	0.025*
**Adverse reactions**	45.83(37.5–50.00)	41.67(25.0–58.33)	0.737	100.00(100.0–100.0)	100.00(100–100)	0.500	100.00(95.83–100.0)	100.00(100.0–100.0)	0.018*
**Treatment satisfaction**	51.39(47.22–56.94)	51.39(51.39–54.51)	0.281	83.33(66.67–100.0)	62.50(55.20–62.50)	0.000**	100.00(95.83–100.0)	66.67(62.50–92.71)	0.000**
**Total score**	211.51(182.45–248.87)	201.51(173.63–229.52)	0.067	484.81±52.91	432.49±34.99	0.000**	544.29(5509.99–568.04)	494.33(455.97–530.15)	0.000**

* P<0.05

**P<0.01

**Table 4:** Multiple linear regression of quality of life between two groups(6 months after discharge).

	β	SE	*t*	*P* Value	95% CI
**A**	709.292	71.450	9.927	0.000	(567.862, 850.722)**
**Group**	-53.575	8.975	-5.969	0.000	(-71.341, -35.808)**
**Age**	-2.507	0.923	-2.715	0.008	-(4.334, -0.679)**
**Sex**	-3.819	11.760	-0.325	0.746	(-27.097, 19.459)
**Marital status**	-12.029	12.121	-0.992	0.323	(-36.022, 11.965)
**Education level**					
**Junior high school**					
**Senior high school**	2.690	14.639	0.184	0.854	(-26.286, 31.667)
**College**	-31.933	22.806	-1.400	0.164	(-77.075, 13.210)
**Income**					
**<1000**					
**1000~2000**	7.613	16.533	0.460	0.646	(-25.113, 40.338)
**>2000**	28.841	24.298	1.187	0.238	(-19.256, 76.937)
**Comorbidity**	-6.190	11.228	-0.551	0.582	(-28.416, 16.036)
**Smoking**					
**Never**					
**Before**	8.889	15.744	0.565	0.573	(-22.276, 40.054)
**Yes**	15.467	16.546	0.935	0.352	(-17.286, 48.219)
**Endovascular Stents**					
**1**					
**2**	3.634	11.284	0.322	0.748	(-18.702, 25.971)
**≥3**	-11.088	12.992	-0.854	0.395	(-36.804, 14.628)

**P<0.01

### Comparison of clinical parameters between two groups of participants

Table 5 shows a comparison of the two groups in terms of clinical indicators. The analysis indicated that there were significant differences between the groups 6 months after discharge.

**Table 5:** Comparison of clinical indicators between two groups ($\bar{\text{x}}$±s, Q_2_(Q_1_-Q_3_)).

Variable	Intervention group (n = 70)	Control group (n = 70)	Test Statistics	*P* Value
**SBP**^**a**^	120.83±9.68	126.99±10.15	-4.102	0.000**
**DBP**^**b**^	75(68.0–80.0)	80(70.0–86.25)	3.526	0.000**
**TC**^**a**^	3.26±0.83	3.97±0.71	-3.033	0.002**
**TG**^**b**^	1.44(1.09–1.93)	1.88(1.20–2.26)	2.841	0.004**
**HDL cholesterol**^**b**^	1.10(0.98–1.29)	1.51(1.20–1.90)	5.781	0.000**
**LDL cholesterol**^**b**^	1.84(1.29–2.14)	2.00(1.80–2.61)	3.005	0.003**
**BMI**^**a**^	23.30±2.71	24.44±3.48	-2.072	0.042*
**LVEF**^**a**^	56.88±7.64	50.68±9.4	5.310	0.000**

^a^Independent t-test was used to compare the means of two groups for a normally distributed data

^b^Mann-Whitney U test was used to compare the medians of two groups for a skewed data

SBP = Systolic blood pressure. DBP = Diastolic blood pressure. TC = Total cholesterol. TG = Triglyceride. HDL cholesterol = High-density lipoprotein cholesterol. LDL cholesterol = Low-density lipoprotein cholesterol. BMI = Body mass index. LVEF = Left ventricular ejection fraction

*P<0.05

**P<0.01

### Comparison of rates of cardiovascular events and readmission

**Fig 2:**
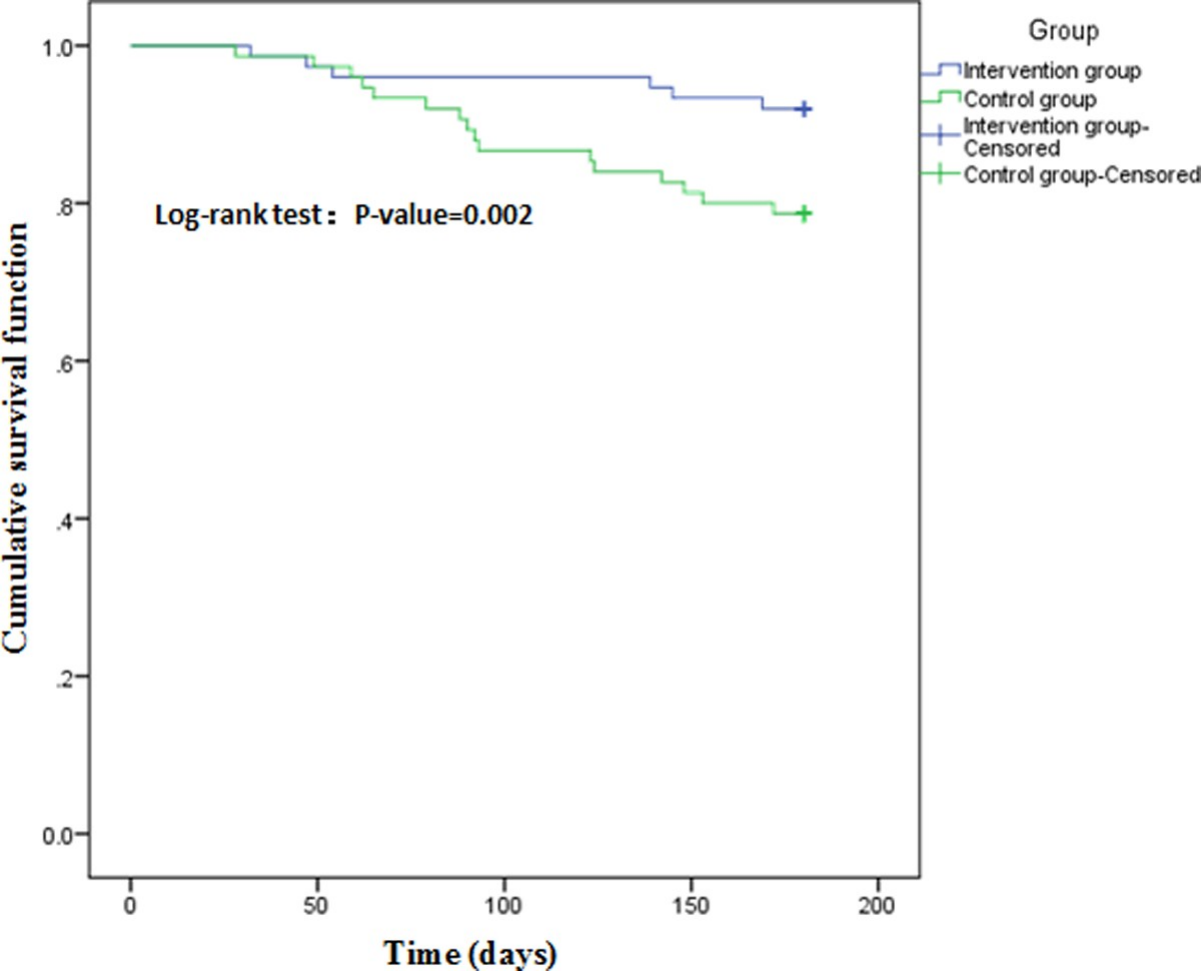
Comparing cumulative risk during follow-up for 6 months in two groups.

During the 6-month follow-up period, 22 cardiovascular events occurred in the two groups, and 10 patients were lost to follow-up (Table 6). The cardiovascular event rate in the intervention group was 8.0% (6 patients) and 21.3% (16 patients) in the control group 6 months after discharge (P<0.05). The log-rank test and Cox proportional hazards model were used to analyze the survival time associated with noncardiovascular events. Univariate and multivariate Cox proportional hazard regression are shown in Tables 7 and 8. Group (whether transitional health management was carried out in elderly patients with acute myocardial infarction undergoing PCI) (HR = 2.837, 95%CI = 1.004–8.014), education level (HR = 9.415, 95%CI = 1.445–61.364) and income (>2000, HR = 0.075, 95%CI = 0.013–0.415) were independent risk factors for survival. In Fig 2, patients in the control group had a higher incidence of cardiovascular events than those in the intervention group (χ^2^ = 5.237, P = 0.022).

**Table 6:** Comparison of cardiovascular events between the two groups (n%).

Cardiovascular events	Before the intervention		1 month after discharge		6 months after discharge	
	Intervention group (n = 75)	Control group (n = 75)	Intervention group (n = 75)	Control group (n = 75)	Intervention group (n = 75)	Control group (n = 75)
**Cardiogenic death**	0	0	0	0	0 (0)	1 (1.4)
**Recurrent angina**	0	0	1	1	3 (4.0)	8 (11.4)
**Recurrent myocardial infarction**	0	0	0	0	1 (1.3)	1 (1.4)
**Arrhythmias**	0	0	0	0	2 (2.7)	5 (7.1)
**Heart failure**	0	0	0	0	0 (0)	1 (1.4)
**χ**^**2**^	0.000		0.000		5.393	
***P* Value**	1.0		1.0		0.020*	

* P<0.05

**Table 7:** Univariate Cox proportional hazard regression analysis of adverse cardiovascular events after PCI.

Characteristic	β	SE	Wald	*P* Value	Crude HR	95% CI
**Group**	-1.053	0.479	4.834	0.028*	0.349	(0.136, 0.892)
**Age**	-0.019	0.037	0.261	0.609	0.981	(0.912, 1.055)
**Sex**	0.513	0.434	1.399	0.237	1.670	(0.714, 3.908)
**Marital status**	0.426	0.443	0.924	0.336	1.531	(0.642, 3.651)
**Education level**						
**Junior high school**			1.789	0.409	1.0	1.00
**Senior high school**	0.627	0.516	1.474	0.225	1.872	(0.680, 5.152)
**College**	0.573	0.753	0.579	0.447	1.773	(0.405, 7.755)
**Income**						
**<1000**			7.401	0.025*	1.0	1.00
**1000~2000**	-0.910	0.521	3.048	0.081	0.402	(0.145, 1.118)
**>2000**	-1.968	0.731	7.252	0.007*	0.140	(0.033, 0.585)
**Comorbidity**	-0.730	0.443	2.708	0.100	0.482	(0.202, 1.150)
**Smoking**						
**Never**			0.491	0.782	1.0	1.00
**Before**	-0.410	0.612	0.447	0.504	0.664	(0.200, 2.205.)
**Yes**	-0.363	0.592	0.376	0.540	0.696	(0.218, 2.218)
**Endovascular Stents**						
**1**			3.884	0.143	1.0	1.00
**2**	0.735	0.563	1.706	0.192	2.086	(0.692, 6.284)
**≥3**	-0.337	0.764	0.195	0.659	0.714	(0.160, 3.190)

* P<0.05

**Table 8:** Multivariate Cox proportional hazard regression analysis of adverse cardiovascular events after PCI.

Characteristic	β	SE	Wald	P Value	Adjusted HR	95% CI
**Group**	1.043	0.530	3.874	0.048*	2.837	(1.004, 8.014)
**Age**	-0.017	0.042	0.163	0.687	0.983	(0.905, 1.068)
**Sex**	0.113	0.533	0.045	0.832	1.119	(0.394, 3.180)
**Marital status**	0.531	0.547	0.944	0.331	1.701	(0.582, 4.968)
**Education level**						
**Junior high school**			7.275	0.026*	1.0	1.00
**Senior high school**	1.206	0.645	3.494	0.062	3.341	(0.943, 11.840)
**College**	2.242	0.956	5.497	0.019*	9.415	(1.445, 61.364)
**Income**						
**<1000**			8.926	0.012*	1.0	1.00
**1000~2000**	-1.106	0.600	3.404	0.065	0.331	(0.102, 1.071)
**>2000**	-2.594	0.875	8.784	0.003*	0.075	(0.013, 0.415)
**Comorbidity**	-0.669	0.504	1.761	0.184	0.512	(0.191, 1.376)
**Smoking**						
**Never**			1.102	0.576	1.0	1.00
**Before**	-0.708	0.714	0.985	0.321	0.492	(0.122, 1.994)
**Yes**	-0.741	0.767	0.932	0.334	0477	(0.106, 2.415)
**Endovascular Stents**						
**1**			5.410	0.067	1.0	1.00
**2**	0.907	0.602	2.268	0.132	2.477	(0.761, 8.067)
**≥3**	-0.443	0.807	0.302	0.583	0.642	(0.132, 3.120)

* P<0.05

The chi-square test was used to compare the readmission rate for cardiovascular disease within 6 months after discharge in the two groups (Table 9). The analysis showed that the number of rehospitalizations in the intervention group was significantly lower than that in the control group (P<0.05), indicating that transitional health management significantly reduced the number of readmissions for patients with cardiovascular disease.

**Table 9:** Comparison of readmission rates between two groups (n/%).

Variable	Intervention group (n = 70)	Control group (n = 70)	χ^2^	P Value
**Readmission rate**	2(2.9%)	8(11.4%)	3.877	0.049*

* P<0.05

## Discussion

The results of this study showed that patients in the intervention group were treated with medications through a three-month transitional health management program for elderly patients with acute myocardial infarction that included the issuance and interpretation of management manuals, one-on-one assessments, education, supervision, and implementation. The review and healthy lifestyle compliance rates were significantly higher in the intervention group than in the control group (Table 2). There was no significant difference in exercise compliance between the control and the intervention groups (Table 2). This could be because the average age of the study population was more than 65 years old (Table 1), and the subjects may have been worried that getting an exercise-induced injury or increasing the amount of exercise would increase the heart load[29].

In addition, our results showed that there was a statistically significant difference in the scores between the two groups at 1 month and 6 months after discharge in terms of quality of life, especially at 6 months (Table 3). The improvement in quality of life in the intervention group was better than that in the control group, indicating that these programs benefit patients during the transition period after PCI. The link between the rehabilitation level and prognosis is inseparable. The positive intervention effect of combined health management on clinical indicators in our study is the reduction in clinical indicators after the intervention (all P<0.05, Tables 4 and 5). This is a useful and exciting message that the patients’ rehabilitation progressively improved with the assistance of the researchers. This finding is also in line with the European Heart Association publications. In 2015, the European Heart Association[30] pointed out that the essence of coronary artery disease management is to estimate the incidence of cardiovascular disease in the future. Blood pressure classification and blood lipids have a strong linear relationship with the incidence of coronary heart disease. At the same time, they are associated with coronary atherosclerosis.

Our findings also indicated that the risk of combined cardiovascular events in the intervention group was significantly lower than that in the control group (Tables 6, 7 and 8 and Fig 2), with the clear benefit occurring at one and a half months during the intervention period. Furthermore, the rate of readmission in the intervention group was also lower than in the control group (Table 9).

PCI treatment in patients with acute myocardial infarction does not mean that the disease has been cured because no treatment can reverse the pathological process of coronary atherosclerosis nor can it eliminate cardiovascular risk factors[9]. Some studies[10] pointed out that the recurrence of adverse cardiovascular events after PCI in patients with acute myocardial infarction is a high-probability event and that the prognosis of patients with acute myocardial infarction is closely related to treatment compliance and their quality of life after discharge. Although adherence to coronary heart disease treatment has improved in recent years, it is still not ideal[31]. Kerzner et al.[32] pointed out that only 25% to 40% of patients undergoing PCI strictly use their medications after discharge. The WHO[33] investigated 218,047 patients with postmyocardial infarction in many countries and pointed out that more than 50% did not strictly follow the doctors’ advice. Noncompliance with doctors’ advice has become a global problem. At present, the strengthening of health management during the transition period has been carried out in various fields abroad[34,35,36]. Jackson1 et al.[37] performed transitional period health management in patients with coronary heart disease, which significantly reduced the rate of secondary hospital admissions. The majority of patients with acute myocardial infarction in China are discharged after 3–5 days of stable disease, and then they return to their families for follow-up treatment and rehabilitation[38]. Therefore, it is very important and meaningful to implement transitional health management for elderly patients with myocardial infarction undergoing PCI treatment. The program can not only accurately understand the patients’ difficulties in a timely fashion during the transition period after discharge but also provide guidance and support to patients.

Compared to the rich and varied forms of health education available abroad, such as cardiac rehabilitation institutions or full-scale health management practitioners, Chinese patients can only receive limited medical education from the hospital when they are discharged[36]. The patients often fail to completely comprehend the information, which contributes to their inability to successfully transition to recovery. Particularly for older patients with physical and cognitive decline, postdischarge management needs to be strengthened to consolidate treatment effects, improve prognosis, and reduce medical costs.

## Conclusion

The transitional health management intervention, including 12 weeks follow-up, succeeded in elderly patients with AMI undergoing PCI, a period when the majority of patients experience the transition period from hospital to family for rehabilitation. The results demonstrated an improved outcome of participants assigned to a health management intervention group. The intervention provided comprehensive and systematical guide for elderly patients and helped patients adopt active and positive coping styles gradually in the transition period. It ameliorated patient compliance and quality of life, restored the stability of clinical indicators. The ultimate effect of intervention is to help patients make a successful transition to health.

## Supporting information

S1 File CONSORT flowchart. (DOC)

S2 File CONSORT 2010 checklist. (DOC)

S3 File Handbook of transitional health management after PCI. (PDF)

S4 File Protocol±Chinese copy. (DOC)

S5 File Protocol±English copy. (DOCX)

S6 File Table-quality of life. (DOC)

S7 File Date. (XLS)
