# Supplementary material for: Effects of transitional health management on adherence and prognosis in elderly patients with acute myocardial infarction in percutaneous coronary intervention: A cluster randomized controlled trial
Source: PLoS One. 2019 May 31;14(5):e0217535. doi: 10.1371/journal.pone.0217535 (PMC6544260; doi:10.1371/journal.pone.0217535)
Supplement: S1 File — (DOC) [file pone.0217535.s001.doc]

**
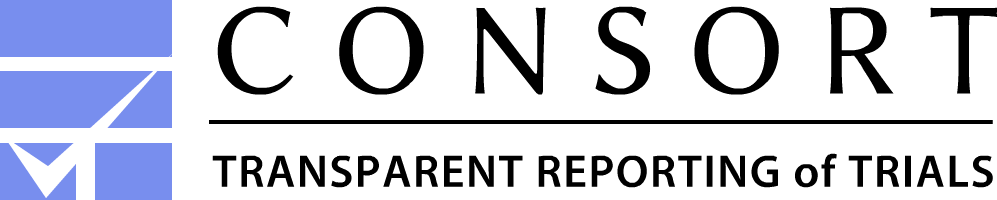
**

**CONSORT 2010 Flow Diagram**

**Allocation**

**Analysis**

**Follow-Up**

**Enrollment**

Assessed for eligibility (n=318)

Excluded (n=168)

♦  Not meeting inclusion criteria (n=99)

♦  Declined to participate (n=69)

♦  Other reasons (n= 0 )

Analysed (n=70)
♦ Excluded from analysis (give reasons) (n=0 )

Lost to follow-up (Lever Suzhou) (n=3)

Discontinued intervention (Family refused experiment) (n=2)

Allocated to intervention (n=75)

♦ Received allocated intervention (n=75)

♦ Did not receive allocated intervention (give reasons) (n= 0 )

Lost to follow-up (withdrew consent prior to delivery) (n= 2)

Discontinued intervention (no answered the phone five times) (n=3)

Allocated to intervention (n=75)

♦ Received allocated intervention (n=75)

♦ Did not receive allocated intervention (give reasons) (n=0 )

Analysed (n=70)
♦ Excluded from analysis (give reasons) (n= 0)

Randomized (n= 150 )
