# Supplementary material for: Effects of transitional health management on adherence and prognosis in elderly patients with acute myocardial infarction in percutaneous coronary intervention: A cluster randomized controlled trial
Source: PLoS One. 2019 May 31;14(5):e0217535. doi: 10.1371/journal.pone.0217535 (PMC6544260; doi:10.1371/journal.pone.0217535)
Supplement: S3 File — (PDF) [file pone.0217535.s003.pdf]

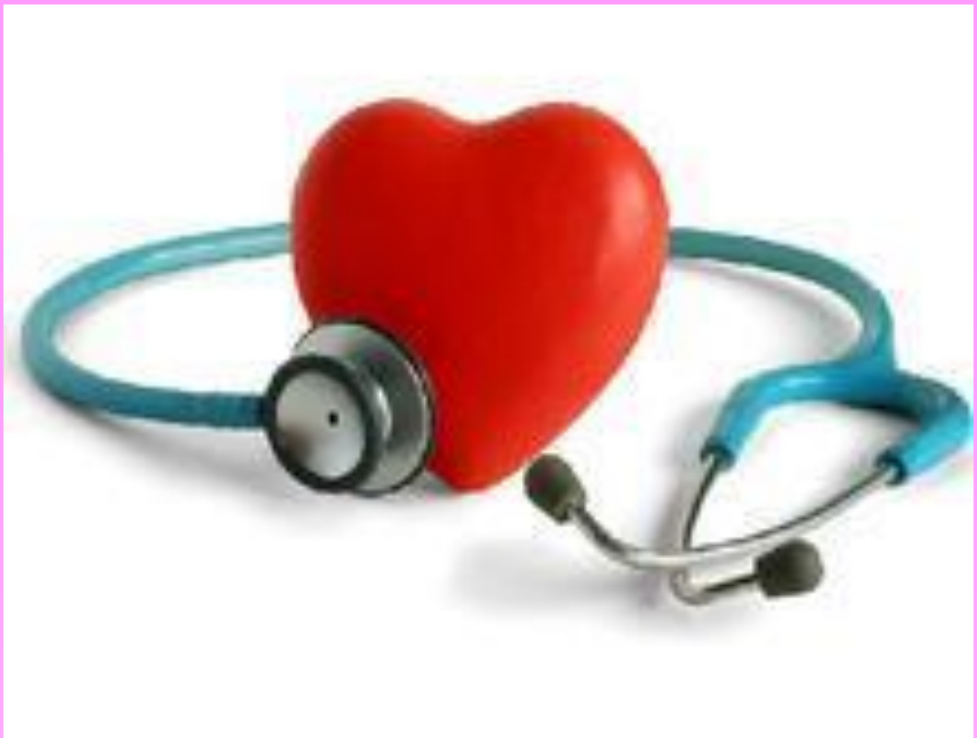

冠脉内支架植入后过渡期

健康教育手册

苏州大学附属第一医院 心血管内科

2016年4月编制

目录 CONTESTS

- 冠心病的形成……………………………1
- 冠心病介入疗法…………………………2
- 出院了，还要注意什么……………3
- 支架术后为何要服药………………4
- 心梗后需要终身服药吗……………5
- 为什么需要复查…………………………6
- 出院后如何选择食物………………7
- 出院后如何做运动……………………8
- 冠心病不可不防…………………………9
- 其他注意事项……………………………11

冠心病的形成

您知道吗

1. 正常健康的冠状动脉

- 血管内壁表面平滑
- 血管壁纤维富弹性，血液运行顺畅，令心肌得到足够的氧气及营养

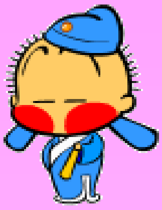

“我很健康诶”

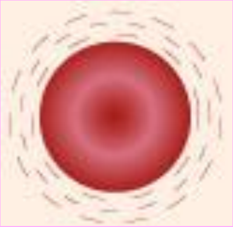

2. 亚健康状态的冠状动脉

- 血管管道收缩
- 血管壁纤维富弹性，血液运行顺畅，令心肌得不到足够的氧气及营养

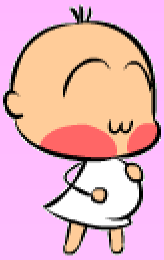

“我有点难受”

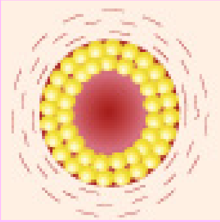

3. 被阻塞的冠状动脉

- 血管管道闭塞，血液不能流通，心肌因缺乏氧气及营养而坏死

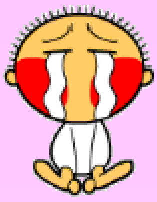

“快救救我啊”

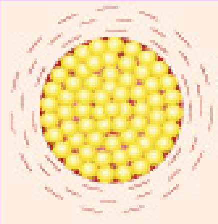

什么是冠脉介入

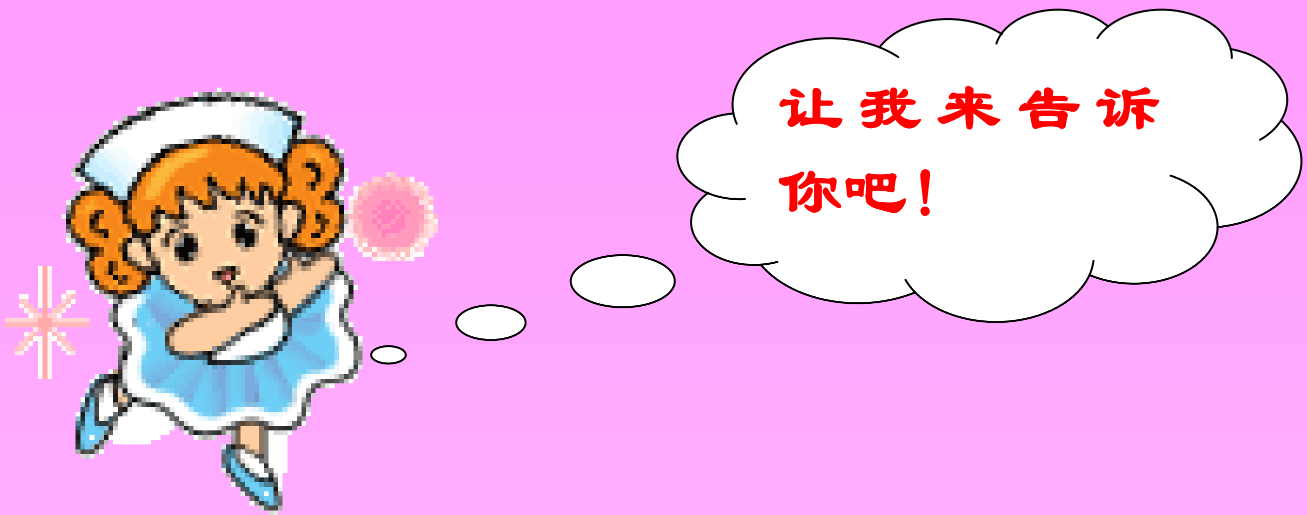

介入治疗的**基本原理**是将球囊导管通过血管穿刺置入狭窄的血管内，在体外将球囊加压膨胀，撑开狭窄的血管壁，使病变血管恢复畅通。这一技术应用于人体冠脉，可**保证**冠脉的**通畅**，增加了心肌的**血供**。

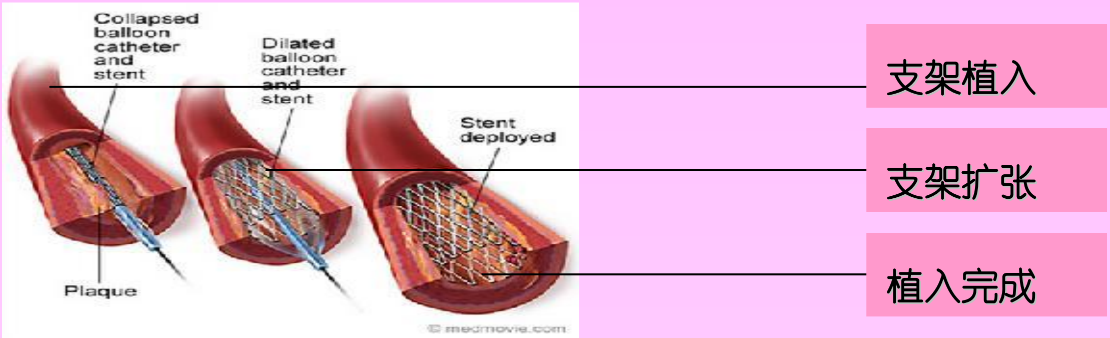

出院了，还要注意什么

要

合理膳食，适量运动，戒烟限酒，心理平衡

加

管住嘴，适量多运动，控制血压、血脂、血糖

油

生活规律，改变不良生活方式，戒烟戒酒，饮食清淡，低盐、低脂（少食用荤油）低糖。控制主食量，切忌暴饮暴食。日常八分饱。进行适当强度的体力活动

哦

冠心病、高血压、糖尿病、高脂血症等为终生性疾病，要终生服药控制，定期来门诊复查，严格按时按量服药，切忌自行增减药量

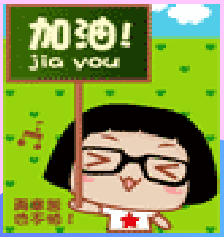

支架术后为何要服药

- 支架只能治好或者暂时支撑病变部位的血管，对其他血管没有治疗作用；
- 术后服药第一是防止支架内长血栓，第二是防止没放支架的血管动脉硬化继续恶化；
- 防止支架内长血栓的药物有阿司匹林、波立维或泰嘉，防止冠心病其他部位血管病变进展的药物有他汀类降血脂药物；
- 为了治疗和控制好高血压、糖尿病；
- 药物可以去除影响血管病变的不利因素，达到控制冠心病其他部位血管病变的目的。

心梗后需要终身服药吗

让我来告诉你

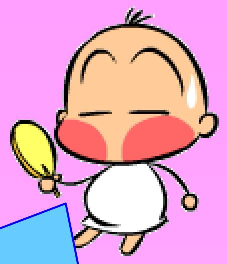

心梗后，心肌供血不同往常，供血不足容易诱发心绞痛，甚至再发心梗。应根据复查时病情及时调整用药，除非出现必须停止服用的其他疾病。比如波立维目前主张至少服用 1 年以上，如果病情稳定无须终身服用，但经济条件可以的话适当延长服用时间也可以。而硝酸酯类只要没有胸闷胸痛等症状可以不服用。

为什么需要复查

介入支架仅处理直径狭窄超过70%的血管，没有处理轻中度的病变，仍然有再次发生心绞痛、心肌梗死的可能。

成功介入治疗存在一定的复发率，已经疏通的血管仍然有再次狭窄的可能

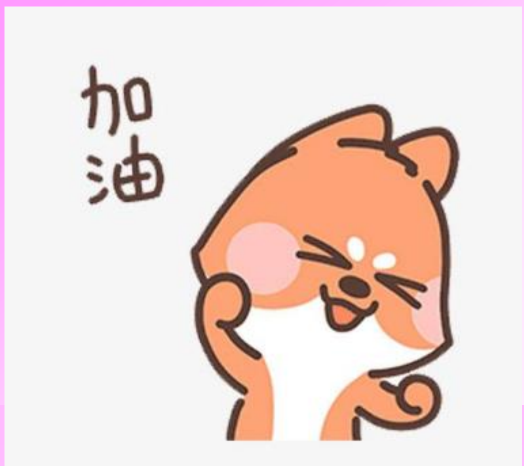

合并糖尿病、多支冠脉病变和存在临界病变患者，复查可以评估疗效以指导今后的治疗策略。

[illegible]何选择食物

高脂肪 ×
高胆固醇 ×

动物脂肪、动物内脏 ×
肥肉 ×
贝壳类 ×
糖、烟、酒、巧克力 ×

“少食或忌食”

瘦肉、鱼类✓
植物油✓
奶类、鸡蛋✓

“适量就行啦”

富含维生素✓
食物纤维 ✓
无机盐✓
微量元素 ✓
谷类、豆制品✓
菌藻类✓
瓜类水果✓
茶叶✓

“可以随意进食哦”

出院后如何做运动

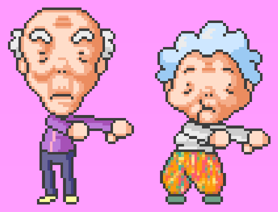

术后几天内，如经股动脉做的，要避免下肢活动度过大，经桡动脉做的，要避免上肢用力，以防出血。

平时运动应在下午进行；如有晨运习惯，建议早起洗刷后先喝一杯温水，5 分钟后才去晨运，切忌激烈。

运动强度：心率的控制在 180-年龄。如为 60 岁的人，应该控制在 180-60=120 次/分以下，运动至微微出汗即可。

不要从事令心情波动太大的工作。

冠心病不可不防哦

——冠心病的危险因素（一）

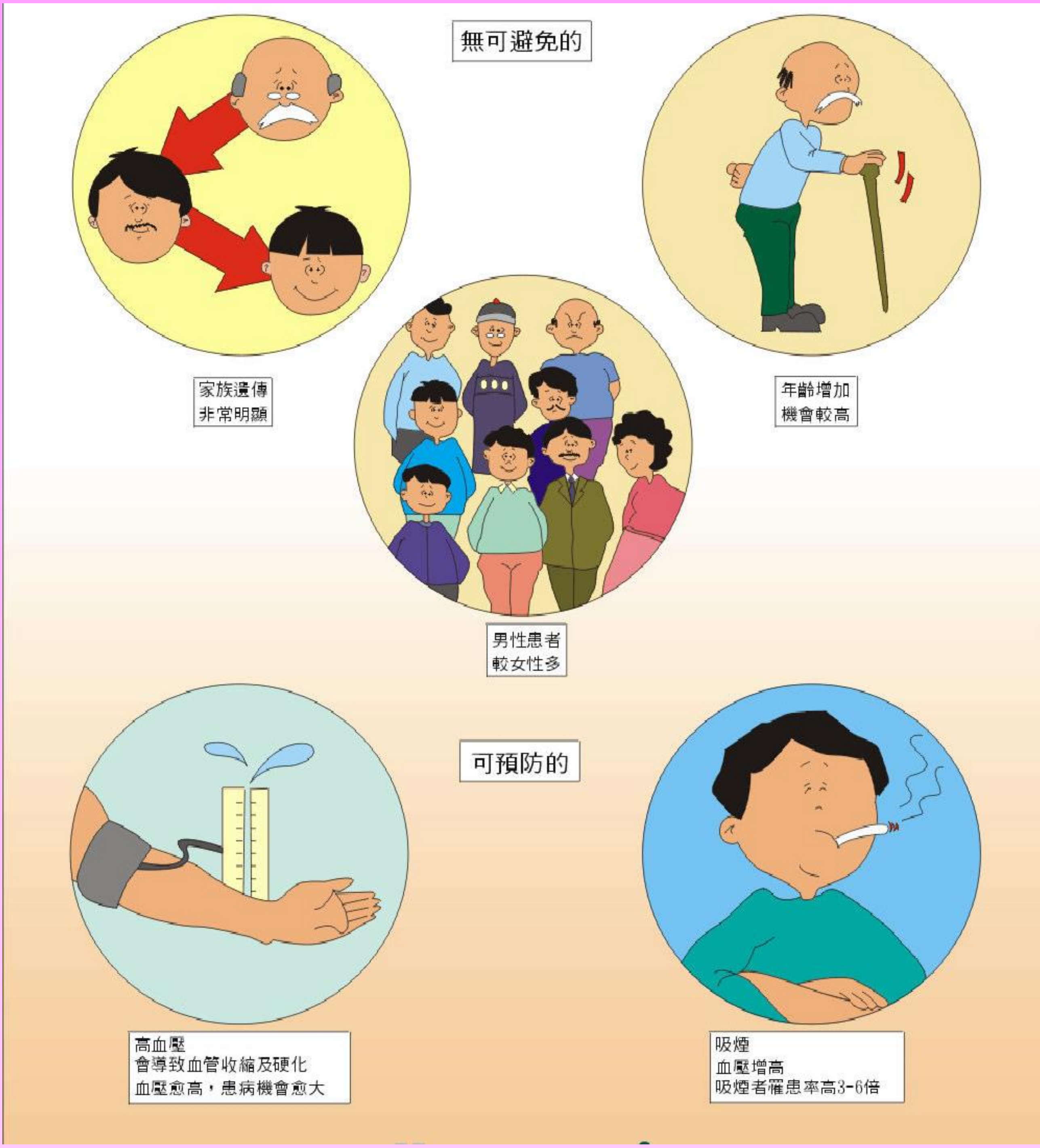

冠心病不可不防哦

——冠心病的危险因素（二）

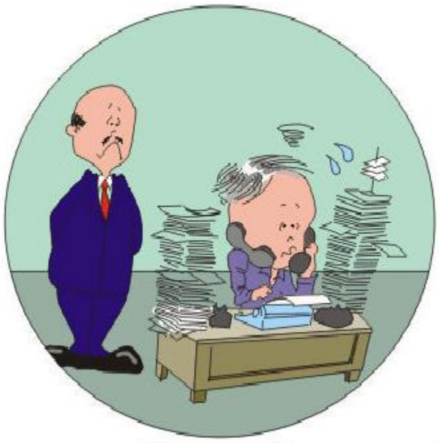

生活緊張
- 令血壓增高
- 增加發病率

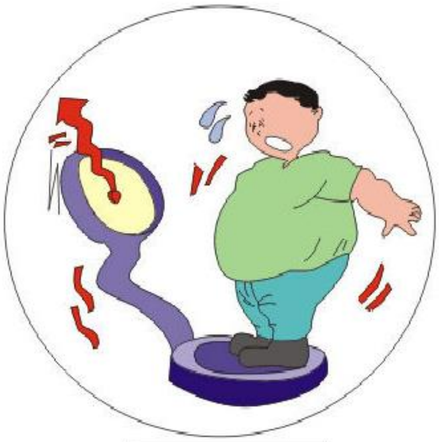

過胖
- 心臟負擔增加
- 胖子罹患率高2倍

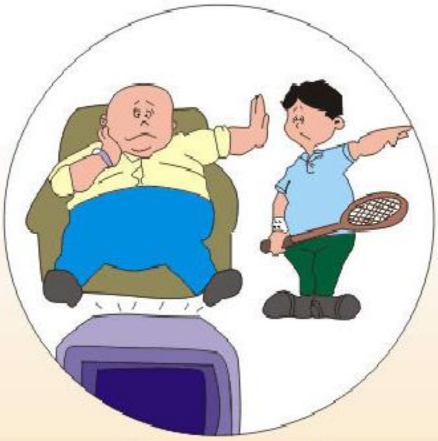

缺乏運動
- 令心肌逐漸衰退
- 罹患率增2.5倍

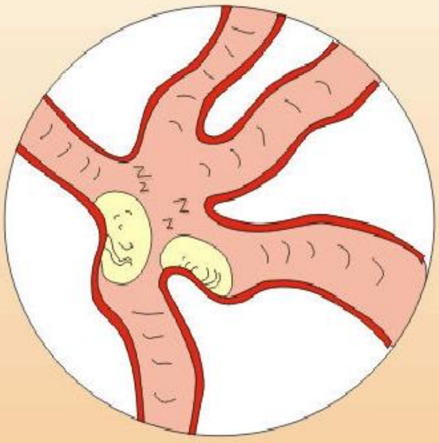

血內膽固醇過高
- 膽固醇沉澱於血管內壁造成動脈粥樣硬化。

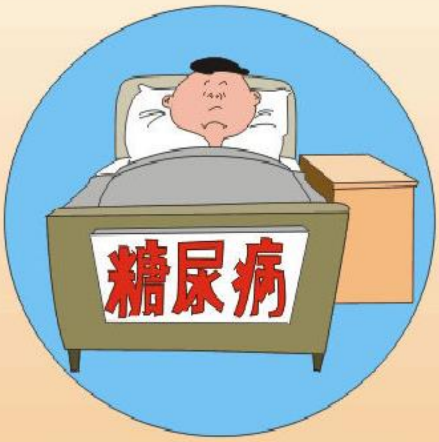

糖尿病
- 可引發血管病變尤其是冠狀動脈

其他注意事项（一）

我的情况	应对方式
急性胸痛发作	保持镇静，停止一切活动，就地休息。<br>立即舌下含服硝酸甘油 1 片或速效救心丸 10 粒。如无效有可能发生了急性心肌梗死，应马上拨打急救电话，尽快去医院。如自行服药缓解，也应尽快去医院复查冠脉造影。<br>如心绞痛发作症状和术前相似，有支架内再狭窄的可能。<br>如症状与术前不同，可能有新发冠脉病变。
感冒、发热、腹泻	冠脉介入术后的药物和抗感冒药、退热药和治疗腹泻的药物一般没有冲突。<br>感冒、发热和腹泻时，血容量往往不足，应根据当时血压情况减少或停用降压药物，以免造成血压过度的降低。（最好询问医生后）<br>倍他乐克、阿替洛尔等 β 受体阻滞剂尽量不要停用。
外　伤	如因为疼痛和紧张等因素诱发心绞痛，治疗药物和急性发作时一样。<br>如为体表局部出血，可压迫止血，不要停用阿司匹林和氯吡格雷等抗凝药物。<br>如果怀疑有内脏出血或颅内出血，必需停用阿司匹林和氯吡格雷并尽快入院，在严密观察下治疗。

其他注意事项（二）

我的情况	应对方式
外出旅游	外出时应自备急救的药盒如硝酸甘油、速效救心丸等和日常的口服药。<br>尤其是介入术后服用的阿司匹林和氯吡格雷，千万不能忘记。<br>外出旅游饮食起居经常不规律，但一定要按时服药。<br>有心绞痛发作时如口含药物不能缓解，应立刻前往当地最近的医院就医。
做重要事情前	遇到重要事情前，经常会因为精神紧张而导致血压升高，心率加快。为避免由此引发的心绞痛，可在重要事情前根据当时的血压和心率情况 加服消心痛 2-4 片、倍他乐克半片或 1 片。
夫妻生活	夫妻生活对体力的消耗很大，介入术后如支架没有再狭窄，冠脉没有新发病变等情况，应不会影响正常的夫妻生活。<br>应根据自身条件酌情进行。<br>如担心会诱发心绞痛，可事先服用消心痛和倍他乐克，对预防心绞痛的发作会有效。

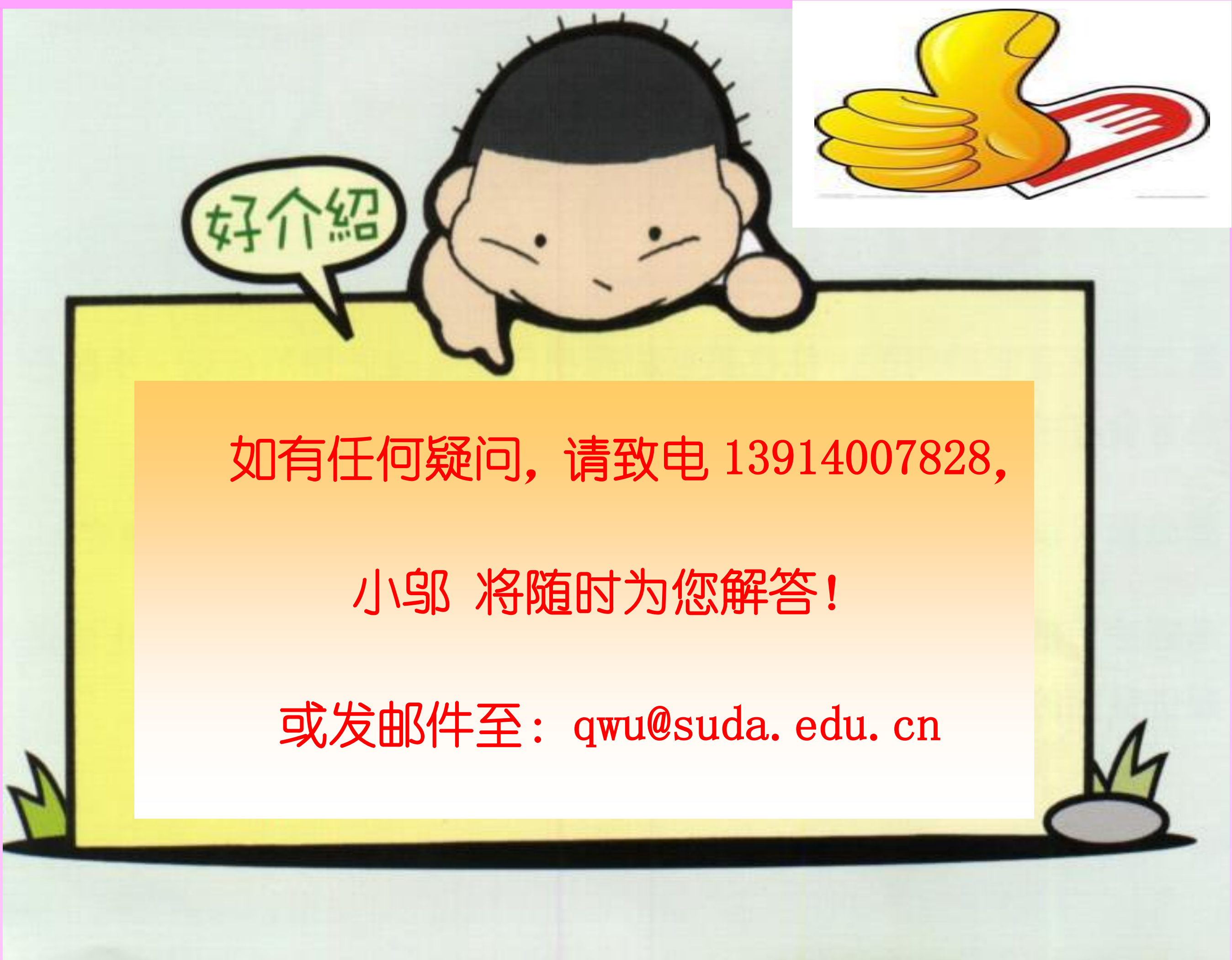

如有任何疑问，请致电 13914007828，

小邬 将随时为您解答！

或发邮件至：qwu@suda.edu.cn
