# Supplementary material for: Effects of transitional health management on adherence and prognosis in elderly patients with acute myocardial infarction in percutaneous coronary intervention: A cluster randomized controlled trial
Source: PLoS One. 2019 May 31;14(5):e0217535. doi: 10.1371/journal.pone.0217535 (PMC6544260; doi:10.1371/journal.pone.0217535)
Supplement: S4 File — (DOC) [file pone.0217535.s004.doc]

版本号： 1.0

版本定稿时间：2016 年05 月20 日

**研究方案简介**

1. 研究背景

近年来，为了提高医疗资源的利用率，患者住院时间明显缩短，往往需要出院后转到基层医院、社区诊所或家庭继续进行治疗和护理，即经历了从一种状态、形式、活动转变到另外一种状态、形式、活动的过渡过程。在这过渡阶段患者仍然有很高的健康照护需求。过渡期护理模式（Transitional Care Model，TCM）是指当患者在疾病治疗与康复阶段，由于诊疗环境和护理需求的改变，需要在各级医疗卫生保健机构之间进行转运与过渡，过渡期护士(Transitional Care Nurse．TCN)为确保患者在这一过渡期间护理工作的协调与连续而采取一系列相应的护理行为。冠心病是严重危害人类健康的常见病。WHO 2000年报告：全球1700万人死于心血管疾病，占各种原因死亡的三分之一。2007年中国卫生部最新流行病学调查显示心血管疾病仅次于恶性肿瘤，高居疾病死亡率第二位，其中冠心病占心血管疾病死亡率的67.1％，平均住院费用也列于各种内科疾病之首，预计到2020年，心血管疾病的危害性将跃升为第一位，成为我国最重要的公共卫生问题之一。冠脉内支架植入术解决了冠状动脉狭窄及血管供血功能，但不可避免地使心肌发生暂时地的急性缺血，心肌泵血功能下降，尤其是术后3个月内心功能下降最明显。因此，对冠脉内支架植入患者出院后实施科学的过渡期护理，在保证患者治疗效果及生存质量等方面是非常重要的。

2. 研究目标

本研究旨在对冠脉后支架植入术后患者实施过渡期护理模式，探讨通过此方法可以提高患者用药、复查等依从性，改善冠脉内支架植入术后患者生存质量，降低再入院率，以充分扩展该病的综合治疗手段，提高临床疗效，减少患者家庭经济负担，降低社会医疗保险的投入。

3. 研究方法

本研究拟对140例（根据Gpower 3.1软件计算样本量）冠脉支架植入术后患者实施以TCM为依据的护理干预。使用SPSS程序生成的随机数字表将研究对象分为干预组70例及对照组70例。干预组在常规出院指导基础上，给予个性化的过渡期护理服务，对照组接受常规出院指导，出院后进行电话随访，解答其疑问。两组于手术后第2天收集患者的基线资料，包括一般资料、依从性、生存质量、血脂水平；出院后2周、1个月、3个月对依从性进行测量；出院后1个月、3个月评价生存质量、血压、体重指数；出院后3个月统计血脂及再入院率。最后进行组内、组间差异的对比观察。本研究结束后将给予对照组相同的护理干预指导。

（1）研究时间：2016年06月01日至2016年12月31日。

（2）研究场所：苏州大学附属第一医院行冠脉内支架植入术治疗的冠心病患者。

（3）样本量：运用Gpower 3.1软件计算样本量，以*α*=0.05，effect size为中等，*t* =0.5和Power=0.8来计算，初步计算本研究需要128例研究对象，考虑到样本失访率，拟取样140例。

（4）纳入标准：

1）初次接受冠脉内支架植入术且手术成功者；

2）苏州市区常住人口或术后至少在三个月内不会离开苏州市区人口；

3）无合并其他严重疾病(如肿瘤、尿毒症等)；

4）神志清，思维和语言能力正常并自愿配合调查。

（5）排除标准：

1）因冠脉狭窄复发而进行二次或三次冠脉支架置入术的患者；

2）心脏扩大、左心收缩功能不全患者；

3）随访期间出现影响其生存的其他脏器严重疾病及恶性肿瘤患者。

4. 观察指标

（1）依从性问卷：采用北京协和医院刘焱老师自行编制的冠脉介入术后患者治疗依从性问卷，该依从性问卷经过效度和信度的测试，得出的内容效度为O.85，重测信度Kappa系数为0.81。评定内容包括服药依从性、复查依从性、生活方式依从性三个方面。

（2）生存质量量表：，采用伦敦大学SaraSchroter教授编制，重庆医科大学曹松梅翻译成中文的适用于评价冠脉介入治疗后生存质量的疾病专有量表CROQ-PTCA-Post。问卷为自评式，包括47个条目6个维度，分别为症状、躯体功能、心理社会功能、认知功能、治疗满意程度、不良反应。

5. 统计处理

采用EpiData 3.1软件建立数据库并进行数据录入、核对，统计数据和分析采用SPSS16.0软件包处理。计量资料以均数±标准差表示，计数资料以百分率(％)表示。两组的人口学资料、各观测指标中的计数资料比较用卡方检验，两组间各观测指标中的计量资料比较用t检验；变量资料的单因素分析，如符合正态者采用Pearson相关分析，非正态者及分类变量采用Spearman秩相关分析；多因素分析时将单因素分析中有统计学意义的变量纳入，采用多元逐步回归分析（*α*_入_＝0.05，*α*_出_＝0.01）确定此模式对患者依从性、生存质量、血脂、血压、体重指数、再入院率的效果。

6. 拟解决的主要问题

探索接受冠脉内支架植入术患者过渡期的需要并进行个体化护理干预。

2016年5月20日
