# Supplementary material for: Effects of transitional health management on adherence and prognosis in elderly patients with acute myocardial infarction in percutaneous coronary intervention: A cluster randomized controlled trial
Source: PLoS One. 2019 May 31;14(5):e0217535. doi: 10.1371/journal.pone.0217535 (PMC6544260; doi:10.1371/journal.pone.0217535)
Supplement: S5 File — (DOCX) [file pone.0217535.s005.docx]

**Trial Study Protocol**

**FULL PROTOCOL TITLE**

Effects of transitional health management on adherence and prognosis in elderly patients with acute myocardial infarction in percutaneous coronary intervention: A cluster randomized controlled trial

**Supported by:**

The Suzhou Science and Technology Bureau

**Version 1.0**

**05/20/2016**

**TABLE OF CONTENTS**  Page

**SYNOPSIS**

1. STUDY OBJECTIVES …………………………………………………………3

1.1 Primary Objective

1.2 Secondary Objectives

2. BACKGROUND…………………………………………………………...…..3

2.1 Rationale

2.2 Supporting Data

3. STUDY DESIGN………………………………………………………...……..4

4. SELECTION AND ENROLLMENT OF SUBJECTS ………………...………5

4.1 Inclusion Criteria

4.2 Exclusion Criteria

4.3 Study Enrollment Procedures

5. STUDY INTERVENTIONS …………………………………………….……6

6. CLINICAL AND LABORATORY EVALUATIONS…………………………7

6.1 Schedule of Evaluations

6.2 Special Instructions and Definitions of Evaluations

7. CRITERIA FOR INTERVENTION DISCONTINUATION……………...……9

8. STATISTICAL CONSIDERATIONS …………………………………...……..9

8.1Outcomes

8.2 Sample Size and Accrual

8.3 Data Analyses

9. HUMAN SUBJECTS ………………………………………………………..…10

9.1 Institutional Review Board (IRB) Review and Informed Consent

9.2 Subject Confidentiality

9.3 Study Modification/Discontinuation

10. PUBLICATION OF RESEARCH FINDINGS ………………………………..10

11. REFERENCES ……………………………………………………….………..10

**1 STUDY OBJECTIVES**

1.1 Primary Objective

The experimental treatment will result in better levers of medication adherence, reexamine adherence, lower rates of adverse cardiovascular events compared to the control treatment.

1.2 Secondary Objectives

The experimental treatment will have better healthy lifestyle and clinical indicators and lower re-admission.

**2 BACKGROUND**

2.1 Rationale

Cardiovascular diseases are the number one killer in the world^[1]^. About 17 million people die of cardiovascular disease each year, and 80% of them occur in less developed countries^[2]^. It is expected that by 2020, the danger of cardiovascular disease will rise to the first place and become one of the most important public health problems in China^[3]^. Among them, coronary heart disease is the most common type, accounting for 67.1% of the death rate of cardiovascular diseases^[4]^. The average cost of hospitalization is also the head of all medical diseases.

In recent years, along with social and economic developments, the ageing of the society has accelerated, and the incidence of acute myocardial infarction (AMI) in the elderly has increased year by year^[5]^. Percutaneous coronary intervention (PCI) can maximize the restoration of coronary blood flow and promote the recovery of cardiac function^[6]^, as well as more and more elderly patients with coronary heart disease select interventional therapy. Many studies have shown that PCI have the advantages of minimal trauma, fast recovery and less complications^[7,8]^.Every year the increasing rate of number of patients undergoing PCI is 30%-40% In China^[9]^. PCI has been widely considered as a treatment measure which is safe and effective^[10]^.

However, most of patients with acute myocardial infarction are discharged in 1 to 2 weeks when they are very stable in China^[11]^. The elderly have the characteristics of old age, physiological deterioration, and many complications, if they do not strengthen health management after PCI, there is still the possibility of coronary restenosis. It is common that people stop medication completely or take less frequently than prescribed^[12]^. Furthermore, studies have shown that it has been estimated that about one third of patients do not adhere to blood pressure or lipid-lowering treatment prescribed following a myocardial infarction (secondary prevention^)[13,14]^.

2.2 Supporting Data

The studies^[14]^ have shown that, 81.7% of the patients think that after discharge, they still hope the medical staff provide relevant services. This indicates that most of the patients hope to get the care and follow-up from the hospital after they leave the hospital^[15]^. Additionally, an intervention using an adolescent peer support group was also found to be an effective way to improve adherence to ART and improve self-image, with previously fearful adolescents perceived to become more confident and outgoing^[16]^.

**3 STUDY DESIGN**

This study will use a single-blind (patient), randomized, controlled method to perform a 3-month transitional health management program for patients undergoing successful coronary stent implantation. Therefore, the goal of our study is to determine whether the health management intervention was able to improve the compliance in the treatment and clinical indicators, reduce cardiovascular events.

Study design

Ethical review

Clinical trial registration

Screening

Informed consents

The experimental treatment

The control treatment

Follow up

Health management intervention program

Standard nursing care

Answered CROQ-PTCA-Post and analyzed at 1 month discharge

Answered CROQ-PTCA-Post and analyzed at 1 month discharge

Answered CROQ-PTCA-Post, compliance questionnaire and analyzed at 6 month discharge

Answered CROQ-PTCA-Post, compliance questionnaire and analyzed at 6 month discharge

Data Analyses

**4 SELECTION AND ENROLLMENT OF SUBJECTS**

4.1 Inclusion Criteria

4.1.1 The patients have been diagnosed with myocardial infarction, have stable vital signs and are arranged for PCI for the first time.

4.1.2 The patients are aged 60 years or more.

4.1.3 The patients have the ability to communicate sufficiently (in Mandarin) to understand the education program.

4.1.4 The patients have no other serious complications, which would interfere with the program (e.g., tumor, uremia).

4.2 Exclusion Criteria

4.2.1 The patients suffer from mental illnesses or cognitively impaired.

4.2.2 Inability or unwillingness of subject or legal guardian/representative to give writ-ten informed consent.

4.3 Study Enrollment Procedures

4.3.1 The participants will be recruited and screened from the two cardiovascular inpatient departments of Teaching Hospital in Suzhou, from June 2016 to December 2016. This study will be registered at [http://www.chictr.org.cn](http://www.chictr.org.cn/edit.aspx?pid=28366&htm=4).

4.3.2 The participants will be numbered in the order of visit and divided into the intervention group and the control group by the SPSS program. To avoid sample contamination (two groups may communicate each other in the same ward), one of the subjects will be adjusted into the intervention group when the intervention group and the control group are in the same ward. Next time one of the examples is adjusted into control group^[20]^.

4.3.3 All study participants will sign informed consents.

4.3.4 All questionnaires are distributed by the same person one by one. Once there are doubt about the questionnaires, an unbiased interpretation would be given by the investigator.

**5 STUDY INTERVENTIONS**

On the basis of consulting relevant literature at home and abroad, the research team will complete the first draft of the “Handbook of Transitional Health Management after PCI”(S6 File). Then, the two deputy chief physicians of cardiovascular medicine and two experienced health management professionals reviewe and revise the draft after 3 rounds, it is the second draft. Finally, five patients without dyslexia and in accordance with the criteria included in the study will be invited to read and give corrections to complete the final draft. The handbook will be distributed to patients and their families in the intervention group on the first postoperative day after PCI and explained one by one, repeatedly emphasizing the precautions during the transition period. After the discharge, two follow-up methods will be adopted: telephone and home visit. Each patient is established a health management file, developed a personalized intervention outline and follow-up timetable. The follow-up content includes: assessing the patient's learning needs; emphasizing the importance of maintaining a good lifestyle, and helping patients to strengthen their self-management skills, such as quitting smoking and drinking, regular work and rest, less salt, less oil and lighter diet; using aerobic exercises such as walking and swimming; drug guidance; urge regular review and so on. Follow-up time: 3 months for a total of 12 weeks. According to the Ebbinghaus Forgetting Curve (Fast, Slow, Slow) feature ^[13]^, the telephone is followed up once a day after discharge. If there are no new problems occurred for 3 days, the phone is changed to 2 times a week for 1 month. Then follow-up is carried out once a month until 12 weeks. If the patients have new problems during follow-up, it means that the above intervention would be repeated for up to 12 weeks. Follow-up visits are performed in the second week after discharge and do not exceed 30 minutes. Telephone follow-up is controlled within 10 minutes. During the follow-up we will answer the patient's doubts and evaluate the effectiveness of the intervention. For different patients, the rehabilitation processes of the disease are not the same and constantly changing. Therefore, the plan will be adjusted according to the actual situation to ensure that the measures will be put in place.

**6 CLINICAL EVALUATIONS**

6.1 Schedule of Evaluations

| **Evaluation** | **Pre- Screening** | **Screening** | **Pre-Entry** | **Entry** | **4wk** | **24** |
| --- | --- | --- | --- | --- | --- | --- |
| Ethical review | X |  |  |  |  |  |
| Informed Consent |  | X |  |  |  |  |
| Clinical Assessment |  |  | X |  |  |  |
| Baseline data |  |  |  | X |  |  |
| Treatment compliance |  |  |  | X |  | X |
| Quality of Life |  |  |  | X | X | X |
| Clinical indicators |  |  |  | X |  | X |
| Cardiovascular events |  |  |  |  |  | X |
| Re-admission |  |  |  |  |  | X |

6.2 Special Instructions and Definitions of Evaluations

The demographic data (age, gender, marital status, education level, income) and clinical characteristics (number of diseased vessels, complications, smoking status, blood pressure, blood lipids, body mass index, left ventricular ejection fraction) are collected as baseline data and 6 month after discharge. Participants in both the intervention- and the control group respond to CROQ-PTCA-Post three times and treatment compliance questionnaire two times either electronically or in print (>70% in Chinese) over the course of the study.

Quality of Life is measured using the CROQ-PTCA-Post assessment. The CROQ-PTCA-Post was established by Sara Schroter in 2004^[22]^. The Chinese version of CROQ-PTCA-Post used in this study was translated by Songmei Cao^[23]^. It is composed of 47 self-reported items, 6 dimensions. The dimensions are symptoms, somatic function, psychosocial function, cognitive function, satisfaction with treatment, and adverse reactions. The Chinese version has been validated, and the Cronbach's alpha coefficient of the dimensions was 0.80～0.94, the test-retest reliability was 0.83～0.94.The content validity was 0.8^[23]^.

Treatment compliance questionnaire. It was provided by Liu Yan, Beijing Union Medical College Hospital, it contains 14 items covering three dimensions, medication compliance, review compliance and lifestyle compliance^[24]^. The Chinese version has been validated and t the Cronbach's alpha coefficient of the dimensions was 0.85, the test-retest reliability was 0.81^[24]^.

**7 CRITERIA FOR INTERVENTION DISCONTINUATION**

If the patients have other serious diseases (such as renal insufficiency, cerebral hemorrhage, etc.) during the study, or if the patient and family members are reluctant to continue participating in the experiment, we will discontinue the follow-up.

**8 STATISTICAL CONSIDERATIONS**

8.1 Outcomes

8.1.1 Primary outcome (including definition)

Treatment compliance questionnaire: It covers three dimensions, medication compliance, review compliance and lifestyle compliance. Rates of cardiovascular events: It covers cardiogenic death,recurrent angina,recurrent myocardial infarction,arrhythmias,heart failure.

8.1.2 Secondary outcomes

Quality of Life.It is composed of 6 dimensions: symptoms, somatic function, psychosocial function, cognitive function, satisfaction with treatment, and adverse reactions.

8.2 Sample Size and Accrual

The sample size estimation is carried out using G*Power 3.1.9.2 software of two population means formula^[18]^. According to other similar studies, the effect rates of the control group and the intervention group are calculated with 0.447 effect size, at 5% significance level, power of 0.8. This means 128 of participants are needed for each group to participate in this study. However, to retain 20% of potential attrition, we should take into an additional number of participants. Therefore, the sample size is expanded to 70 in each group.

8.3 Data Analyses

Data are analyzed using the PASW Statistic 17.0 Programme (SPSS Inc.: Chicago, IL, USA) by two master graduate students who was blinded to the allocation. Measured data are expressed as mean±standard deviation (±s). The t-test is used for the comparison between groups. Comparisons between groups are performed using the Chi-square test or ANOVA for categorical variables. The survival analysis is performed using the Log-rank test. For all analyses, missing data are transformed by mean imputation^[25]^.Throughout the analysis, a p-value of less than 0.05 is considered statistically significant.

**9 HUMAN SUBJECTS**

9.1 Ethics Committee and Informed Consent

This protocol and the informed consent document and any subsequent modifications will be reviewed and approved by the ethics committee responsible for oversight of the study. A signed consent form will be obtained from the subject. For subjects who consent for themselves must sign the consent form; additional-ly, the subject's assent must also be obtained if he or she is able to understand the nature, significance, and risks associated with the study. The consent form will describe the pur-pose of the study, the procedures to be followed, and the risks and benefits of participa-tion. A copy of the consent form will be given to the subject, or legal guardian, and this fact will be documented in the subject’s record.

9.2 Subject Confidentiality

All dates, evaluation forms, reports,and other records that leave the site will bemaintained confidentiality. All records will be kept in a locked file cabinet. Clinical infor-mation will not be released without written permission of the subject, except as the sponsor, or the sponsor’s designee.

9.3 Study Modification/Discontinuation

The study may be modified or discontinued at any time by the sponsor as part of their duties to en-sure that research subjects are protected.

**10 PUBLICATION OF RESEARCH FINDINGS**

Publication of the results of this trial will be governed by the policies and procedures de-veloped by the Executive Committee. Any presentation, abstract, or manuscript will be made available for review by the sponsor to submission.

**11 REFERENCES**

[1][Bansilal S](https://www.ncbi.nlm.nih.gov/pubmed/?term=Bansilal%20S%5BAuthor%5D&cauthor=true&cauthor_uid=26747389),[Castellano JM](https://www.ncbi.nlm.nih.gov/pubmed/?term=Castellano%20JM%5BAuthor%5D&cauthor=true&cauthor_uid=26747389), [Fuster V](https://www.ncbi.nlm.nih.gov/pubmed/?term=Fuster%20V%5BAuthor%5D&cauthor=true&cauthor_uid=26747389).Global burden of CVD: focus on secondary prevention of cardiovascular disease.[Int J Cardiol.](https://www.ncbi.nlm.nih.gov/pubmed/26747389)2015; 201:S1-7.<https://doi.org/10.1016/S0167-5273(15)31026-3>PMID: 26747389.

[2]Ajay VS, Tian M,Chen H,Wu Y,Li X,Dunzhu D,et al.A cluster-randomized controlled trial to evaluate the effects of a simplified cardiovascular management program in Tibet, China and Haryana, India: study design and rationale.BMC Public Health.2014;14:924. <https://doi.org/10.1186/1471-2458-14-924> PMID: 25194850; PubMed Central PMCID: PMC4180354.

[3]Zhang XW,Chen YD.The Role and Value of ST2 Factor in Early Diagnosis and Prognosis of Cardiovascular Diseases.Chinese Journal of Health Medicine.2017;19(3):272-274.

[4]Wang ZH.Psychological intervention in patients with coronary heart disease.Chinese pharmacoeconomics.2014;10 (4):112-113.

[5]Laake K,Seljeflot I,Schmidt EB,Myhre P,Tveit A,Arnesen H,et al.Arnesen HSerum Fatty Acids, Traditional Risk Factors, and Comorbidity as Related to Myocardial Injury in an Elderly Population with Acute Myocardial Infarction.J Lipids.2016;2016:4945720.<http://dx.doi.org/10.1155/2016/4945720> PMID: 26989512. PMCID: PMC4775818.

[6]Yang JH,Hahn JY,Song PS,Song YB,Choi SH,Choi JH,et al.Percutaneous Coronary Intervention for Nonculprit Vessels in Cardiogenic Shock Complicating ST-Segment Elevation Acute Myocardial Infarction[J].Critical Care Medicine.2014;42(1):17-25. https://doi.org/[10.1097/CCM.0b013e3182a2701d](https://doi.org/10.1097/CCM.0b013e3182a2701d) PMID: 24105454.

[7] Zink T,Kralewski J,Dowd B.The Transition of Primary Care Group Practices to Next Generation Models: Satisfaction of Staff, Clinicians, and Patients.J Am Board Fam Med.2017;30(1):16-24. https://doi.org/[10.3122/jabfm.2017.01.160118](https://doi.org/10.3122/jabfm.2017.01.160118) PMID: 28062813.

[8]Liu ZH.Clinical evaluation of real-time exercise therapy in patients with coronary heart disease after PCI.Chinese Journal of Gerontology.2015;35(20):5765-5766.

[9] Li MW,Zhang BL,Zheng X,Chen F,Li R,Shen M.Risk factor score for predicting in-hospital adverse cardiovascular events in elderly patients undergoing percutaneous coronary intervention. Academic Journal of Second Military Medical University.2015;36(8):851-857.

[10]Ahmad Hamdi AH,Dali AF, Mat Nuri TH,Saleh MS,Ajmi NN,Neoh CF,et al.Safety and Effectiveness of Bivalirudin in Patients Undergoing Percutaneous Coronary Intervention: A Systematic Review and Meta-Analysis.Front Pharmacol.Front Pharmacol.2017;8:410.<https://doi.org/10.3389/fphar.2017.00410>PMID:[28744215](https://www.ncbi.nlm.nih.gov/pubmed/28744215). PubMed Central PMCID: PMC5504279.

[11]Chen SQ,Wang Y,Zhang HS,Su P,Xu YS.Application of Transitional Nursing in Recovery of Hand Function of Severed Finger Replantation.Chinese journal of nursing education,2010;7(10):457-459.

[12]Morrison VL,Holmes EA, Parveen S, Plumpton CO, Clyne W,De Geest S,et al. Predictors of self-reported adherence to antihypertensive medicines: a multinational, cross-sectional survey.Value Health.2015 ;18(2):206-216.<https://doi.org/10.1016/j.jval.2014.12.013> PMID:25773556.

[13]Murre JM,Dros J.Replication and Analysis of Ebbinghaus' Forgetting Curve.PLoS One.2015;6;10(7):e0120644.<https://doi.org/10.1371/journal.pone.0120644>PMID:26148023. PubMed Central PMCID:[PMC4492928](https://www.ncbi.nlm.nih.gov/pmc/articles/PMC4492928/).

[14]Yin LZ,Wei Z,Wang QF,Zhong LL,Lv JX. Investigation of Hospitalized Patients' Need for Returning to Family Continuous Nursing Care.Journal of Nursing.2009;24(22):82-83.

[15] Meisinger C, Stollenwerk B,Kirchberger I,Seidl H,Wende R,Kuch B.Effects of a nurse-based case management compared to usual care among aged patients with myocardial infarction: results from the randomized controlled KORINNA study. BMC Geriatr. 2013;13:115. <https://doi.org/10.1186/1471-2318-13-115> PMID: [24168465](https://www.ncbi.nlm.nih.gov/pubmed/24168465).PubMed Central PMCID:PMC3871021.

[16]Fermann GJ,Levy PD,Pang P,Butler J,Ayaz SI, Char D,et al.Design and Rationale of a Randomized Trial of a Care Transition Strategy in Patients With Acute Heart Failure Discharged From the Emergency Department: GUIDED-HF (Get With the Guidelines in Emergency Department Patients With Heart Failure). Circ Heart Fail.2017;10(2): e003581. https://doi.org/10.1161/CIRCHEARTFAILURE.116.003581 PMID: 28188268.

[17]Amberbir A,Singano V,Matengeni A,et al.Dyslipidemia among rural and urban HIV patients in south-east Malawi.PLoS One 2018;13(5):e0197728.

[18]Tillmann AC,Andrade A,Swarowsky A,Guimarães ACA.Brazilian Samba Protocol for Individuals With Parkinson's Disease: A Clinical Non-Randomized Study.JMIR Res Protoc. 2017; 6(7):e129. https://doi.org/[10.2196/resprot.6489](http://doi.org/10.2196/resprot.6489)PMID:[28676466](http://www.ncbi.nlm.nih.gov/pubmed/28676466).PubMed Central PMCID:[5516099](http://www.ncbi.nlm.nih.gov/pmc/articles/5516099).

[19]Ahmad Sharoni SK,Abdul Rahman H, Minhat HS,Shariff-Ghazali S, Azman Ong MH.The effects of self-efficacy enhancing program on foot self-care behaviour of older adults with diabetes: A randomised controlled trial in elderly care facility, Peninsular Malaysia.PLoS One.2018;13(3):e0192417.<https://doi.org/10.1371/journal.pone.0192417>PMID:29534070.PubMed Central PMCID:[PMC5849313](https://www.ncbi.nlm.nih.gov/pmc/articles/PMC5849313/).

[20]Chouinard MC,Robichaud-Ekstrand S.The effectiveness of a nursing inpatient smoking cessation program in individuals with cardiovascular disease.Nurs Res.2005;54(4):243-254.PMID: 16027567.

[21]Sanda B,Vistad I,Sagedal LR,Haakstad LAH,Lohne-Seiler H,Torstveit MK.Effect of a prenatal lifestyle intervention on physical activity level in late pregnancy and the first year postpartum.PLoS One.2017;12(11):e0188102.<https://doi.org/10.1371/journal.pone.0188102>PubMed Central PMID:29176762.PubMed Central PMCID:[PMC5703566](https://www.ncbi.nlm.nih.gov/pmc/articles/PMC5703566/).

[22]Schroter S,Lamping DL.Coronary revascularisation outcome questionnaire (CROQ): development and validation of a new, patient based measure of outcome in coronary bypass surgery and angioplasty.Heart.2004;90(12):1460-1466. <http://dx.doi.org/10.1136/hrt.2003.021899>PMID:15547029.PubMed Central PMCID:[PMC1768578](https://www.ncbi.nlm.nih.gov/pmc/articles/PMC1768578/).

[23]Lv J,Zhang X,Ou S,Gu S,Su Z,Tong S.Influence of Cognitive Behavioral Therapy on Mood and Quality of Life After Stent Implantation in Young and Middle-Aged Patients With Coronary Heart Disease.Int Heart J.2016;57(2):167-172.<https://doi.org/10.1536/ihj.15-259>PMID:26973262.

[24]Liu Y.Correlation analysis of compliance and social support for patients after percutaneous coronary intervention.2006:61-62.Beijing:Peking Union Medical College.

[25]Shen X,Zhu X,Wu Y, Zhou Y,Yang L,Wang Y.Effects of a psychological intervention programme on mental stress, coping style and immune function in percutaneous coronary intervention patients.PLoS One.2018;13(1):e0187745. <https://doi.org/10.1371/journal.pone.0187745>PMID:29357358. PubMed Central PMCID:[PMC5777641](https://www.ncbi.nlm.nih.gov/pmc/articles/PMC5777641/).
