# Supplementary material for: Effects of transitional health management on adherence and prognosis in elderly patients with acute myocardial infarction in percutaneous coronary intervention: A cluster randomized controlled trial
Source: PLoS One. 2019 May 31;14(5):e0217535. doi: 10.1371/journal.pone.0217535 (PMC6544260; doi:10.1371/journal.pone.0217535)
Supplement: S6 File — (DOC) [file pone.0217535.s006.doc]

[**Multiple**](javascript:;) [**linear**](javascript:;) [**regression**](javascript:;) **of quality of life between two groups (1 month after discharge)**

|  | **β** | **SE** | ***t*** | ***P* Value** | **95% CI** |  |
| --- | --- | --- | --- | --- | --- | --- |
| **A** | 620.980 | 60.133 | 10.327 | 0.000 | (501.951, 740.009)** | |
| **Group** | -56.373 | 7.554 | -7.463 | 0.000 | (-71.325, -41.421) ** | |
| **Age** | -2.043 | 0.777 | -2.629 | 0.010 | -0.505* | |
| **Gender** | 2.788 | 9.897 | 0.282 | 0.779 | (-16.803, 22.379) | |
| **Marital status** | -7.848 | 10.201 | -0.769 | 0.443 | (-28.041, 12.346) | |
| **Education level** |  | | | | | |
| **Junior high school** |  |  |  |  |  | |
| **Senior high school** | 9.475 | 12.320 | 0.769 | 0.443 | (-14.912, 33.862) | |
| **College** | -10.540 | 19.194 | -0.549 | 0.584 | (-48.533, 27.452) | |
| **Income** |  | | | | | |
| **<1000** |  |  |  |  |  | |
| **1000～2000** | 22.324 | 13.914 | 1.604 | 0.111 | (-5.218, 49.866) | |
| **2000～3000** | 27.799 | 15.159 | 1.834 | 0.069 | (-2.207, 57.805) | |
| **>3000** | 21.475 | 20.449 | 1.050 | 0.296 | (-19.004,) 61.953 | |
| **Comorbidity** | -10.125 | 9.450 | -1.071 | 0.286 | (-28.830, 8.581) | |
| **Smoker** |  | | | | | |
| **Never** |  |  |  |  |  | |
| **Before** | -8.903 | 13.251 | -0.672 | 0.503 | (-35.132, 17.326) | |
| **Yes** | 5.929 | 13.926 | 0.426 | 0.671 | (-21.636, 33.494) | |
| **Endovascular Stents** |  | | | | | |
| **1** |  |  |  |  |  | |
| **2** | -11.541 | 9.497 | -1.215 | 0.227 | (-30.340, 7.257) | |
| **≥3** | -16.003 | 10.934 | -1.464 | 0.146 | (-37.645,) 5.640 | |

* P＜0.05

**P＜0.01
